# Nutritional status and functional capacity among people living with HIV in routine care: a retrospective cohort study

**DOI:** 10.3389/fpubh.2026.1851353

**Published:** 2026-08-12

**Authors:** Louis Okeibunor Odeigah, Mary Olufunmilayo Ologe, Kazeem Adeola Oshikoya, Joseph Olusesan Fadare

**Affiliations:** 1 Department of Family Medicine, Faculty of Clinical Sciences, College of Medicine and Health Sciences, Afe Babalola University, Ado-Ekiti, Nigeria; 2 Department of Pharmacology and Therapeutics, Faculty of Basic Medical Sciences, College of Health Sciences, University of Ilorin, Ilorin, Nigeria; 3 Department of Pharmacology, Therapeutics and Toxicology, College of Medicine, Lagos State University, Ojo, Nigeria; 4 Department of Medicine/Pharmacology and Therapeutics, College of Medicine, Ekiti State University, Ado-Ekiti, Nigeria

**Keywords:** antiretroviral therapy, body mass index, CD4 count, HIV, nutrition

## Abstract

**Background:**

Malnutrition remains an important concern among people living with HIV (PLHIV), even with widespread antiretroviral therapy (ART). Although ART has improved survival and long-term outcomes, undernutrition persists alongside increasing overweight and obesity, creating a dual nutritional burden that may complicate HIV care. This study described the nutritional status of adults receiving routine HIV care in a tertiary facility in Nigeria.

**Methods:**

We conducted a retrospective cohort study using routinely collected clinical data from the HAART clinic of the University of Ilorin Teaching Hospital, Ilorin, Nigeria. Adults aged ≥ 18 years with complete anthropometric measurements at the index visit were included. For participants with multiple encounters, the index visit was the earliest visit with available body mass index (BMI) data. Nutritional status was classified using standard adult BMI categories. Baseline demographic, anthropometric, treatment, and laboratory characteristics were summarized descriptively. Because laboratory variables had substantial missingness and functional status showed limited variability, adjusted regression analyses were not performed.

**Results:**

Of 1,132 patient records screened, 301 participants met the eligibility criteria. The median age was 52 years [interquartile range (IQR): 45–60 years], and 98.7% were male. Overall, 26 participants (8.6%) were underweight, 165 (54.8%) had normal BMI, 75 (24.9%) were overweight, and 35 (11.6%) were obese. Most participants were receiving tenofovir–lamivudine–dolutegravir (TLD)-based therapy (87.7%). WHO functional status showed no variability; all participants were classified as working. CD4 count and viral load data were available for only 3.7 and 13.3% of participants, respectively.

**Conclusion:**

Undernutrition persists alongside overweight and obesity among adults receiving routine HIV care. The coexistence of nutritional deficits and excess weight supports routine nutritional assessment within long-term HIV care. Given substantial missing laboratory data, lack of functional-status variability, and extreme sex imbalance, findings should be interpreted primarily as descriptive observations.

## 1 Introduction

The widespread scale-up of antiretroviral therapy (ART) has transformed HIV infection into a manageable chronic condition, leading to substantial reductions in HIV-related morbidity and mortality globally. As survival improves, increasing attention has shifted toward long-term health outcomes among people living with HIV (PLHIV), including nutritional status, physical functioning, and overall quality of life (1). Nutrition remains a cornerstone of HIV care, as both undernutrition and overnutrition can adversely affect immune function, treatment response, functional capacity, and the risk of non-communicable diseases.

Historically, HIV infection in sub-Saharan Africa was strongly associated with wasting and severe undernutrition, driven by advanced disease, opportunistic infections, and food insecurity (2). Although the introduction of ART has reduced the prevalence of HIV-associated wasting, undernutrition persists in many settings, particularly among older adults and socioeconomically vulnerable populations (3). Low body mass index (BMI) among PLHIV has been linked to impaired immune recovery, increased hospitalization, and higher mortality, even in the ART era (4).

Concurrently, ART has introduced new nutritional and metabolic challenges. Weight gain, overweight, and obesity have become increasingly prevalent among PLHIV, especially with the use of contemporary regimens such as integrase strand transfer inhibitors (5, 6). This emerging pattern reflects a broader epidemiologic and nutritional transition in low- and middle-income countries, where undernutrition and overnutrition coexist within the same populations and even within the same individuals over time (7). Among PLHIV, this dual burden of malnutrition complicates clinical management and underscores the need for balanced nutritional monitoring.

Functional capacity represents an important but often underexplored dimension of health among PLHIV. Functional status captures an individual’s ability to perform daily activities and reflects the combined effects of nutritional status, comorbid conditions, aging, and chronic inflammation (8). Declines in physical function may occur even in virologically suppressed individuals and may precede overt disability, particularly in aging HIV populations (9). Despite its clinical relevance, functional capacity is infrequently examined alongside nutritional status in routine HIV care, especially in African settings.

In Nigeria, which bears a substantial HIV burden, data on nutritional status and functional capacity among adults receiving long-term ART remain limited. Most existing studies have focused on virologic and immunologic outcomes, with less attention to metabolic and functional health in stable patients engaged in care (10). Understanding the distribution of nutritional status and its relationship with functional capacity and clinical markers in routine care settings is essential for informing comprehensive HIV management strategies.

This study therefore aimed to assess nutritional status and functional capacity among adults living with HIV receiving routine care at a tertiary facility in Nigeria. By describing BMI categories alongside selected sociodemographic characteristics, ART regimen, functional status, and available immunologic and virologic markers, this study provides insight into the evolving nutritional profile of PLHIV in the ART era and highlights areas for targeted intervention.

## 2 Methods

### 2.1 Study design, setting, and data source

This study was a retrospective cohort analysis conducted at the Highly Active Antiretroviral Therapy (HAART) clinic of the University of Ilorin Teaching Hospital (UITH), Ilorin. Data were retrospectively abstracted from individual patient case records and clinical folders maintained as part of routine HIV care. A structured electronic data extraction tool was developed using Kobo Collect to standardize data capture across records. Extracted data were subsequently exported from the Kobo platform into a spreadsheet format for data management and statistical analysis. The final dataset comprised three linked components: the main clinic dataset and separate repeat laboratory datasets for viral load and CD4 measurements. Records across datasets were linked using unique submission identifiers generated by the Kobo tool, which were harmonized into a single patient identifier for analysis.

### 2.2 Data management and quality control

All analyses were conducted in R using a fully scripted workflow to ensure reproducibility. Raw sheets were imported with readxl and standardized variable names using janitor:clean_names(). Date fields were parsed using a robust conversion function that handled native dates, POSIX formats, character dates, and Excel numeric date encodings. Anthropometric plausibility checks were applied *a priori* by flagging implausible values (height < 0.8 m or > 2.2 m; weight < 20 kg or > 250 kg; BMI < 10 or > 80 kg/m^2^) for quality review (flags retained for audit but not used as exclusion criteria unless they resulted in missing/invalid BMI). A missingness summary of key variables was generated and exported for transparency.

### 2.3 Cohort definition and index visit selection

The analytic dataset was restricted to adults aged ≥ 18 years. Pregnant or breastfeeding individuals were excluded to avoid physiologic BMI shifts unrelated to chronic nutritional status. For participants with multiple clinic encounters, a single index visit was selected per person, defined as the earliest visit date with complete BMI (or recalculable BMI from weight and height) and WHO functional status recorded. All primary descriptive analyses were performed at this index visit (one record per patient).

### 2.4 Outcomes

The primary outcome was adult BMI category at the index visit, classified as underweight (< 18.5 kg/m^2^), normal (18.5–24.9 kg/m^2^), overweight (25.0–29.9 kg/m^2^), or obese (≥ 30.0 kg/m^2^). A secondary outcome was underweight prevalence (underweight vs. all other BMI categories combined).

### 2.5 Exposures and covariates

Pre-specified variables of interest included age (years), sex, marital status, occupation group, antiretroviral therapy (ART) regimen group, WHO functional status, CD4 category, and viral suppression status. ART regimens were collapsed into transparent programmatic groupings (TLD; other DTG-based; non-DTG/other; missing). Occupation entries were collapsed into broad groups (e.g., farming, public service, business, homemaker, other, missing) to reduce sparse categories.

CD4 and viral load were treated as clinical variables (not outcomes). CD4 category was defined as ≤ 200, 201–350, or > 350 cells/μL. Viral suppression status was defined using the < 200 copies/mL threshold (suppressed vs. unsuppressed), with “unknown/missing” retained as explicit categories where applicable.

### 2.6 Linkage of laboratory results to index visit

To align repeated laboratory measurements with the index visit, the nearest CD4 value within ±90 days and the nearest viral load value within ±365 days of the index date were selected per participant using a deterministic nearest-neighbor rule (minimum absolute day difference; ties resolved by selecting the most recent result). These windows were chosen to balance clinical relevance and real-world documentation gaps in routine care.

### 2.7 Statistical analysis

Baseline characteristics were summarized overall and stratified by BMI category. Continuous variables were described using median (Q1, Q3), while categorical variables were summarized as frequency and percentage. Underweight prevalence was estimated as the proportion of participants classified as underweight at the index visit. Because CD4 count and viral load data were substantially incomplete and WHO functional status showed no variability, inferential analyses and adjusted regression models were not performed.

Analytic tables and figures were exported as publication-ready outputs using gtsummary, flextable, officer, and ggplot2. Reporting was structured to align with STROBE guidance for observational studies.

### 2.8 Reproducibility

A fixed random seed [set.seed(123)] was used for any procedures that could involve randomness. The analysis script generated an auditable output directory containing: quality-control summaries, the final index-visit analytic dataset, publication tables, figures, and model outputs. Analyses were conducted in R.

## 3 Results

### 3.1 Study population and analytic sample

This retrospective study utilized routinely collected clinical data from the Highly Active Antiretroviral Therapy (HAART) clinic of the University of Ilorin Teaching Hospital (UITH), Ilorin. A total of 1,132 patient records were screened for eligibility. The analytic dataset was restricted to adults aged ≥ 18 years, and pregnant or breastfeeding individuals were excluded because of physiologic changes in body weight and body mass index (BMI) that could influence nutritional classification. To ensure a single observation per participant, records from individuals with multiple clinic encounters were consolidated, and an index visit was defined as the earliest encounter with available BMI and WHO functional status data. Participants lacking the information required to determine BMI or functional status at the index visit were excluded from the final analytic dataset.

Following application of these eligibility criteria and selection procedures, 301 participants were included in the final analytic cohort. All analyses were performed using data from the index visit only. The substantial reduction from the initially screened population reflects the retrospective nature of the study and the requirement for complete anthropometric and functional status information. Consequently, the possibility of selection bias cannot be excluded and should be considered when interpreting the findings.

Availability of laboratory data was limited. CD4 count measurements linked within ±90 days of the index visit were available for 11 participants (3.7%), while viral load measurements linked within ± 365 days of the index visit were available for 40 participants (13.3%) (Table 1). Missing laboratory data accounted for 96.3 and 86.7% of observations for CD4 count and viral load, respectively. Consequently, these variables are presented descriptively to provide clinical context and were not used in inferential analyses.

**Table 1:** Availability of CD4 count and viral load data relative to the index visit.

Laboratory variable	Available (n)	Total participants (N)	Available (%)	Missing (n)	Missing (%)
CD4 count within ±90 days of index visit	11	301	3.7	290	96.3
Viral load within ±365 days of index visit	40	301	13.3	261	86.7

Availability of laboratory data among adults living with HIV included in the analytic cohort. CD4 count was available for only 11 participants (3.7%), while viral load measurements were available for 40 participants (13.3%). Missing laboratory data accounted for 96.3 and 86.7% of observations for CD4 count and viral load, respectively. Owing to the substantial degree of missingness, these variables were reported descriptively and were not included in inferential analyses.

### 3.2 Baseline sociodemographic and clinical characteristics

#### 3.2.1 Overall characteristics

The baseline sociodemographic and clinical characteristics of the 301 participants at the index visit, stratified by BMI category, are summarized in Table 2. The overall median age was 52 years [interquartile range (IQR): 45–60 years]. Most participants were aged 40 years and older, with the largest proportions occurring within the 40–49 years (29.9%), 50–59 years (28.9%), and ≥ 60 years (27.9%) age groups. The cohort was overwhelmingly male, comprising 296 of 301 participants (98.7%), while females accounted for only 1.3%; therefore, sex-specific descriptions are limited and should be interpreted cautiously.

**Table 2:** Baseline sociodemographic, anthropometric, and clinical characteristics of adults living with HIV at the index visit, stratified by BMI category.

Variable	Overall *N* = 301^1^	Underweight *N* = 26^1^	Normal *N* = 165^1^	Overweight *N* = 75^1^	Obese *N* = 35^1^
Age (years)	52.0 (45.0, 60.0)	53.5 (43.0, 63.0)	52.0 (42.0, 61.0)	52.0 (47.0, 59.0)	49.0 (45.0, 58.0)
Age groups					
18–29	17 (5.6%)	3 (11.5%)	11 (6.7%)	2 (2.7%)	1 (2.9%)
30–39	23 (7.6%)	1 (3.8%)	15 (9.1%)	5 (6.7%)	2 (5.7%)
40–49	90 (29.9%)	5 (19.2%)	48 (29.1%)	22 (29.3%)	15 (42.9%)
50–59	87 (28.9%)	8 (30.8%)	40 (24.2%)	28 (37.3%)	11 (31.4%)
≥ 60	84 (27.9%)	9 (34.6%)	51 (30.9%)	18 (24.0%)	6 (17.1%)
Sex					
Female	4 (1.3%)	0 (0.0%)	2 (1.2%)	2 (2.7%)	0 (0.0%)
Male	296 (98.7%)	26 (100.0%)	163 (98.8%)	72 (97.3%)	35 (100.0%)
Unknown	1	0	0	1	0
Marital status					
Married	244 (81.3%)	21 (80.8%)	129 (78.7%)	68 (90.7%)	26 (74.3%)
Single	48 (16.0%)	4 (15.4%)	32 (19.5%)	6 (8.0%)	6 (17.1%)
Widowed	8 (2.7%)	1 (3.8%)	3 (1.8%)	1 (1.3%)	3 (8.6%)
Unknown	1	0	1	0	0
Occupation group					
Business/self-employed	17 (5.6%)	0 (0.0%)	9 (5.5%)	5 (6.7%)	3 (8.6%)
Farming	22 (7.3%)	3 (11.5%)	17 (10.3%)	2 (2.7%)	0 (0.0%)
Homemaker	2 (0.7%)	0 (0.0%)	1 (0.6%)	0 (0.0%)	1 (2.9%)
Missing	0 (0.0%)	0 (0.0%)	0 (0.0%)	0 (0.0%)	0 (0.0%)
Other	142 (47.2%)	15 (57.7%)	77 (46.7%)	34 (45.3%)	16 (45.7%)
Public service	37 (12.3%)	1 (3.8%)	19 (11.5%)	12 (16.0%)	5 (14.3%)
Trading	81 (26.9%)	7 (26.9%)	42 (25.5%)	22 (29.3%)	10 (28.6%)
Weight (kg)	68.0 (60.0, 77.0)	51.0 (50.0, 53.0)	64.0 (58.0, 69.0)	78.0 (71.0, 85.0)	90.0 (83.0, 98.0)
Height (m)	1.7 (1.6, 1.8)	1.7 (1.7, 1.8)	1.7 (1.7, 1.8)	1.7 (1.6, 1.8)	1.6 (1.6, 1.7)
BMI (kg/m^2^)	23.4 (21.4, 27.3)	17.6 (16.1, 18.3)	22.1 (21.1, 23.3)	27.4 (26.1, 28.7)	32.4 (30.4, 34.9)
Regimen group					
TLD	264 (87.7%)	22 (84.6%)	138 (83.6%)	69 (92.0%)	35 (100.0%)
Other DTG-based	3 (1.0%)	0 (0.0%)	2 (1.2%)	1 (1.3%)	0 (0.0%)
Non-DTG/other	34 (11.3%)	4 (15.4%)	25 (15.2%)	5 (6.7%)	0 (0.0%)
Missing	0 (0.0%)	0 (0.0%)	0 (0.0%)	0 (0.0%)	0 (0.0%)
Current TPT	4 (1.3%)	1 (3.8%)	1 (0.6%)	2 (2.7%)	0 (0.0%)

^1^Median (Q1, Q3); n (%); BMI, body mass index; ART, antiretroviral therapy; TLD, tenofovir–lamivudine–dolutegravir; DTG, dolutegravir; TPT, tuberculosis preventive therapy. Baseline sociodemographic, anthropometric, and clinical characteristics of 301 adults living with HIV receiving routine care at the University of Ilorin Teaching Hospital, stratified by body mass index (BMI) category at the index visit. Continuous variables are presented as median [interquartile range (IQR)], while categorical variables are presented as frequency and percentage. WHO functional status is not shown because all participants were classified as working, resulting in no variability. Laboratory variables (CD4 count and viral load) are presented separately in Table 1 owing to substantial missingness and were not included in inferential analyses.

Most participants were married (81.3%), whereas 16.0% were single and 2.7% were widowed. Occupationally, participants were predominantly classified within the “Other” occupation category (47.2%), followed by trading (26.9%), public service (12.3%), farming (7.3%), and business/self-employment (5.6%). Homemakers represented less than 1% of the cohort.

Anthropometric measures varied across BMI categories as expected. The overall median body weight was 68.0 kg (IQR: 60.0–77.0 kg), while the median height was 1.70 m (IQR: 1.64–1.75 m). The overall median BMI was 23.4 kg/m^2^ (IQR: 21.4–27.3 kg/m^2^). Median body weight increased progressively from 51.0 kg among underweight participants to 90.0 kg among obese participants. Similarly, median BMI increased from 17.6 kg/m^2^ in the underweight group to 32.4 kg/m^2^ in the obese group.

Clinically, most participants were receiving tenofovir–lamivudine–dolutegravir (TLD)-based antiretroviral therapy (264/301; 87.7%). Non-DTG-based regimens accounted for 11.3% of participants, while only 1.0% received alternative DTG-based regimens. WHO functional status showed no variability, with all participants classified as working at the index visit.

CD4 count and viral load information were available for only a small subset of participants and are therefore presented descriptively. As shown in Table 1, CD4 measurements within ±90 days of the index visit were available for 11 participants (3.7%), while viral load measurements within ±365 days of the index visit were available for 40 participants (13.3%). Owing to the substantial degree of missing laboratory data, these variables were not used in inferential analyses.

#### 3.2.2 Availability of laboratory data

Availability of laboratory data is summarized in Table 1. CD4 count measurements within ±90 days of the index visit were available for 11 of 301 participants (3.7%), while viral load measurements within ±365 days of the index visit were available for 40 participants (13.3%). Missing laboratory data accounted for 96.3 and 86.7% of observations for CD4 count and viral load, respectively.

Given the substantial degree of missingness, these laboratory variables are presented descriptively to provide clinical context for the cohort. Consequently, no inferential analyses involving CD4 count or viral load were performed, and no conclusions regarding immunologic or virologic correlates of nutritional status can be drawn from the available data.

### 3.3 Nutritional status distribution

#### 3.3.1 BMI categories at index visit

**Figure 1:**
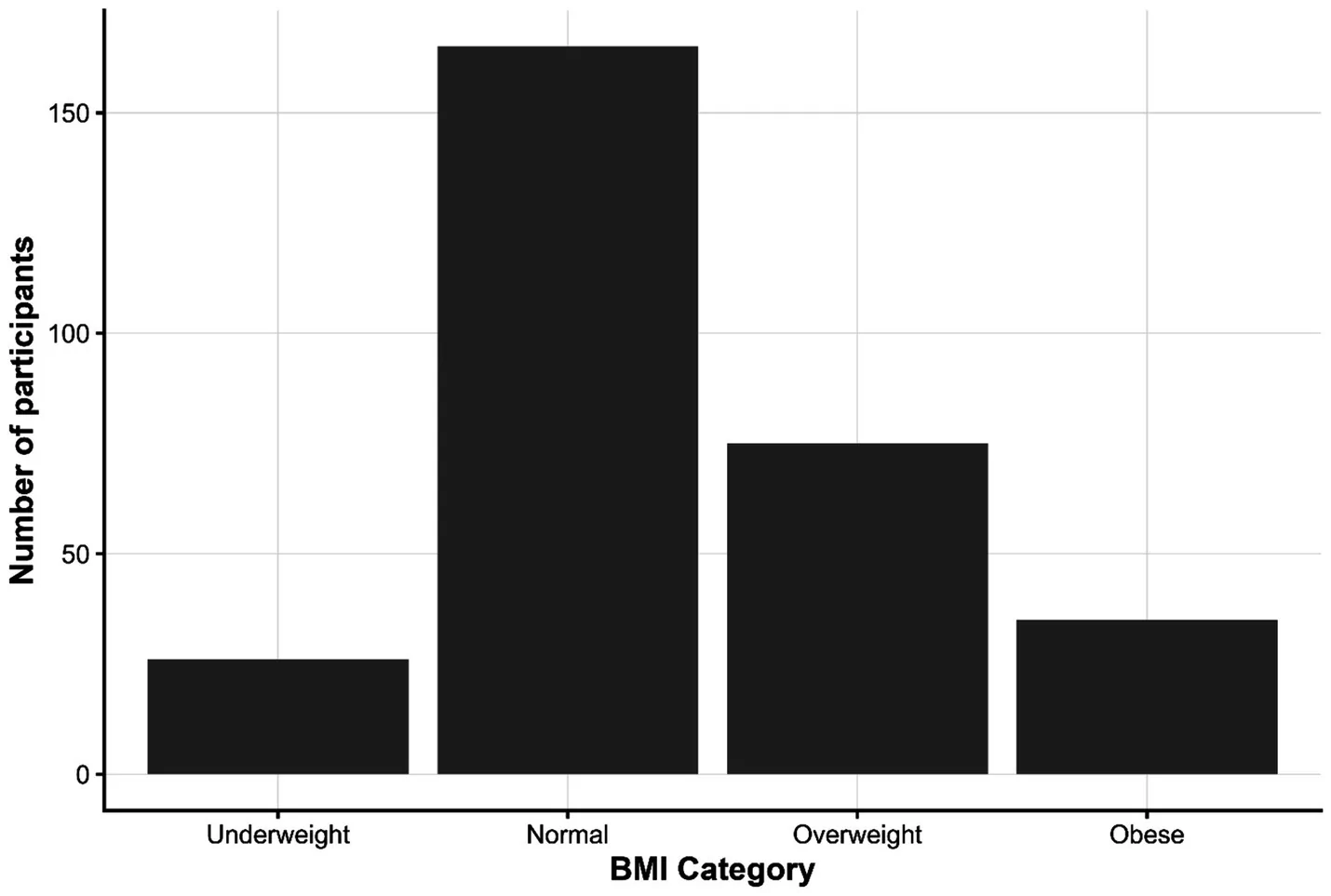
Distribution of BMI categories among adults living with HIV at the index visit. Bar chart showing the distribution of body mass index (BMI) categories among 301 adults living with HIV at the index visit. BMI categories were defined using standard adult cut-offs. Underweight remained present in a subset of participants despite ongoing antiretroviral therapy.

At the index visit, the distribution of BMI categories among the 301 adults living with HIV is shown in Figure 1. The majority of participants were classified as having normal body weight (165/301; 54.8%), followed by overweight (75/301; 24.9%) and obesity (35/301; 11.6%). Underweight was observed in 26 participants, corresponding to a prevalence of 8.6%. Despite widespread ART exposure in this cohort, underweight remained a notable finding, underscoring the persistence of undernutrition among a subset of individuals receiving routine HIV care.

#### 3.3.2 Sex-stratified BMI distribution

**Figure 2:**
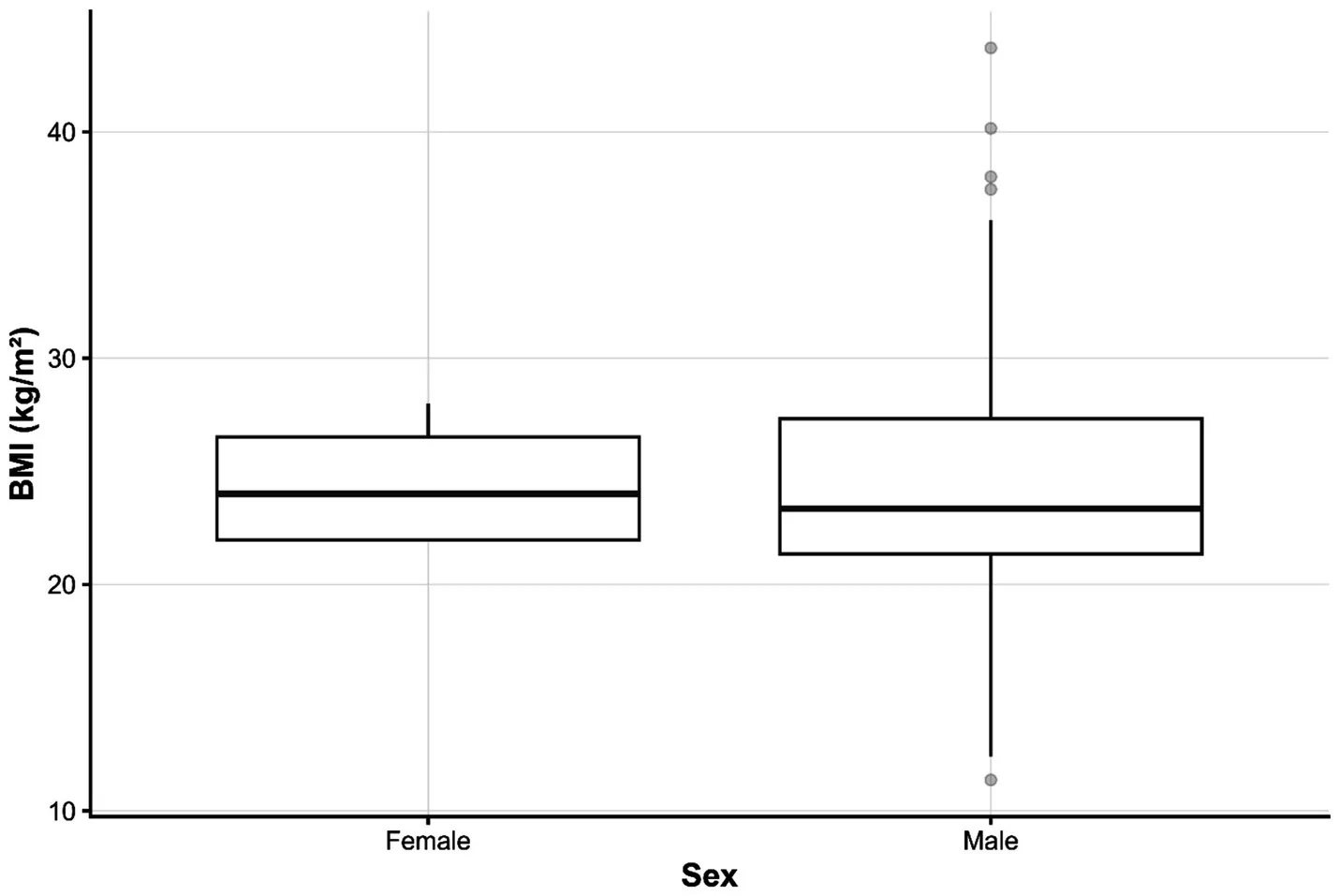
BMI distribution by sex among adults living with HIV at the index visit. Boxplots showing the distribution of body mass index (BMI) by sex among 301 adults living with HIV at the index visit. The horizontal line represents the median BMI, boxes indicate the interquartile range, and whiskers and points denote distribution spread and outliers. Because only four female participants were included, this figure should be interpreted descriptively and not as a formal sex-specific comparison.

BMI distributions stratified by sex at the index visit are shown in Figure 2. Because only four female participants were included, any sex-specific comparison should be interpreted cautiously. The boxplot is therefore presented descriptively and should not be interpreted as evidence of comparable BMI distributions between females and males. The wider spread and outliers observed among male participants largely reflect the overwhelming male predominance of the cohort.

## 4 Discussion

### 4.1 Nutritional status in adults receiving routine HIV care

In this retrospective analysis of adults living with HIV receiving routine care at a tertiary HAART clinic in Nigeria, undernutrition remained present despite widespread antiretroviral therapy exposure and preserved functional capacity. Although the overall prevalence of underweight was relatively modest, its persistence alongside a substantial burden of overweight and obesity highlights a clear dual pattern of malnutrition within this population. This finding reinforces the evolving nutritional landscape of HIV care in sub-Saharan Africa, where improved survival has not uniformly translated into optimal nutritional health.

The presence of underweight among adults with a median age exceeding 50 years is particularly notable. Older adults living with HIV may experience compounded nutritional vulnerability due to age-related sarcopenia, chronic inflammation, altered energy metabolism, and comorbid conditions, even in the context of virologic control and functional stability (11–13). Prior studies have demonstrated that low BMI in people living with HIV is independently associated with increased morbidity, impaired immune recovery, and reduced quality of life, underscoring its clinical relevance beyond virologic outcomes (14, 15).

At the same time, the high prevalence of overweight and obesity observed in this cohort is consistent with growing evidence of weight gain and adiposity among ART-treated populations. Multiple studies across Africa and globally have documented a shift toward overweight and obesity following ART initiation, particularly in the era of integrase strand transfer inhibitor–based regimens such as dolutegravir (5, 16, 17). While ART regimen was not interpreted causally in this study, the predominance of TLD-based therapy provides important clinical context for understanding contemporary BMI distributions among people living with HIV.

The coexistence of underweight and overweight within the same clinic population reflects a dual burden of malnutrition increasingly recognized in chronic HIV care. This pattern mirrors trends observed in the general population in many low- and middle-income countries undergoing nutritional and epidemiologic transition, but is further complicated in people living with HIV by chronic immune activation, metabolic dysregulation, and social vulnerability (18, 19). Importantly, this dual burden challenges HIV programs that have historically focused nutritional interventions primarily on wasting and undernutrition, highlighting the need for more nuanced, individualized nutritional strategies.

Notably, all participants in this cohort were classified as working according to WHO functional status at the index visit, suggesting relative clinical stability and engagement in care. However, the persistence of abnormal BMI across the nutritional spectrum indicates that functional status alone may not adequately capture underlying nutritional or metabolic risk. Similar observations have been reported in other ART-treated cohorts, where individuals may remain functionally active despite significant alterations in body composition and nutritional reserves (20, 21).

Taken together, these findings emphasize that nutritional status remains a critical, independent dimension of health among adults living with HIV receiving routine care. Even in the context of ART scale-up, viral suppression, and preserved functional capacity, both undernutrition and overnutrition continue to coexist. Routine nutritional assessment using simple anthropometric measures such as BMI therefore remains essential, particularly in aging HIV populations. Integrating nutritional monitoring into long-term HIV care may help identify individuals at risk of adverse clinical outcomes and guide timely, context-appropriate interventions.

### 4.2 Functional capacity and nutritional context

WHO functional status showed no variability in the present study, with all participants classified as working at the index visit. Consequently, meaningful assessment of relationships between functional capacity and nutritional status was not possible. Although the uniform classification suggests that participants were generally clinically stable and capable of performing routine daily activities, the absence of variation precluded evaluation of whether functional status differed across BMI categories or nutritional states.

The lack of variability observed in functional status may reflect the relative clinical stability of individuals receiving long-term antiretroviral therapy in routine care settings. Previous studies have demonstrated that adults living with HIV may maintain functional independence despite the presence of nutritional deficits, reduced lean body mass, or age-related declines in physical performance, particularly when HIV infection is effectively managed with ART (22–24). Consequently, broad functional classifications may not adequately capture early manifestations of nutritional vulnerability or subclinical impairments in physical performance.

Similarly, the coexistence of overweight and obesity within a clinically stable HIV population is consistent with reports from contemporary ART-treated cohorts, where excess body weight frequently occurs alongside preserved short-term physical functioning (25, 26). However, increasing evidence suggests that overweight and obesity may contribute to adverse metabolic outcomes, reduced physical performance, frailty, and functional decline over time, particularly among older adults living with HIV (27). Such effects may not be readily identified through routine WHO functional status assessment alone.

An additional consideration is the limited sensitivity of the WHO functional status scale for detecting subtle gradations in physical health. While the scale remains useful for identifying advanced disease and severe disability, it does not assess domains such as muscle strength, exercise tolerance, mobility limitation, or frailty, which have been shown to correlate more closely with nutritional status and body composition (28, 29). More comprehensive measures of physical functioning, including gait speed, grip strength, physical activity assessments, and frailty indices, may therefore provide greater discriminatory value in future studies.

The inability to assess functional capacity represents an important limitation of the present study. Future investigations incorporating more detailed measures of physical performance and functional health may help clarify the relationship between nutritional status and functional outcomes among adults living with HIV, particularly within aging populations receiving long-term ART.

### 4.3 Immunologic and Virologic markers in relation to nutritional status

CD4 count and viral load measurements were included in this study to provide clinical context for the cohort; however, availability of these data was extremely limited. CD4 count measurements within ±90 days of the index visit were available for only 11 participants (3.7%), while viral load measurements within ±365 days were available for 40 participants (13.3%). Consequently, meaningful assessment of relationships between nutritional status and immunologic or virologic markers was not possible, and these variables were not included in inferential analyses.

The limited availability of CD4 count and viral load data represents an important constraint in interpreting the relationship between nutritional status and HIV disease markers. Although immunologic recovery and virologic suppression remain central goals of HIV care, increasing evidence suggests that nutritional status, body composition, and metabolic health may not always parallel traditional markers of HIV disease control, particularly among individuals receiving long-term ART (30, 31). Nutritional abnormalities may persist despite successful immune reconstitution and virologic suppression, reflecting the influence of factors such as aging, dietary practices, socioeconomic conditions, chronic inflammation, and ART-associated metabolic effects (32–34).

The high degree of missing laboratory data observed in this study is also reflective of challenges commonly encountered in retrospective analyses of routine-care datasets. Laboratory monitoring is often performed according to clinical need, resource availability, and programmatic guidelines, resulting in incomplete capture of immunologic and virologic measurements within predefined analytical windows (35). While such missingness limits the ability to draw conclusions regarding associations between nutritional status and HIV disease markers, it highlights the practical value of routinely available clinical measures such as BMI for nutritional surveillance and patient monitoring.

Therefore, the present study cannot provide evidence regarding the relationship between nutritional status and immunologic or virologic outcomes because of the limited availability of CD4 count and viral load data. Future prospective studies with more complete laboratory monitoring will be necessary to clarify the interplay between nutritional status, immune recovery, and virologic control among adults living with HIV receiving long-term ART.

An additional limitation is the reliance on BMI as the sole measure of nutritional status. Although BMI is widely used in clinical and epidemiological research, it does not distinguish between lean and fat mass, assess body composition, identify sarcopenia, or capture central adiposity. Important nutritional indicators such as waist circumference, muscle mass, dietary assessments, and validated nutritional screening tools were unavailable in the routine clinical dataset. Consequently, the nutritional profile described in this study should be interpreted within the constraints of BMI-based classification.

## 5 Conclusion

This retrospective cohort study of adults receiving routine HIV care at a tertiary facility in Nigeria demonstrates that undernutrition persists alongside a substantial burden of overweight and obesity in the contemporary antiretroviral therapy era, highlighting the coexistence of nutritional deficits and excess weight among people living with HIV. Although most participants were receiving tenofovir–lamivudine–dolutegravir (TLD)-based therapy and were classified as functionally active at the index visit, underweight remained present in a subset of participants, while overweight and obesity were common. These findings underscore the importance of incorporating routine nutritional assessment into long-term HIV care, particularly as treated HIV populations continue to age and experience evolving nutritional and metabolic challenges. However, the findings should be interpreted in light of several important limitations, including substantial missingness of CD4 count and viral load data, the absence of variability in WHO functional status, the overwhelming male predominance of the cohort, and the retrospective single-center study design. These limitations restricted the ability to evaluate immunologic, virologic, and functional correlates of nutritional status and preclude causal inference. Consequently, the results should be interpreted primarily as descriptive observations rather than evidence of independent associations. Future prospective, multicenter studies incorporating more comprehensive laboratory monitoring and detailed functional assessments are needed to better characterize the determinants and clinical consequences of nutritional status among adults living with HIV receiving long-term antiretroviral therapy.

## Data Availability

The data analyzed in this study is subject to the following licenses/restrictions: The raw data supporting the conclusions of this article will be made available by the authors, without undue reservation. Requests to access these datasets should be directed to odeigahlo@abuad.edu.ng.
